# Using an Accelerated Undergraduate Needs Finding Course to Build Skills, Inspire Confidence, and Promote Interest in Health Technology Innovation

**DOI:** 10.1007/s43683-023-00109-3

**Published:** 2023-04-10

**Authors:** Lyn Denend, Susie Spielman, Ross Venook, Ravinder D. Pamnani, David Camarillo, James Wall, Joseph Towles

**Affiliations:** 1 Byers Center for Biodesign, Stanford University, 318 Campus Drive, E100, Stanford, CA 94305 USA; 2 Department of Bioengineering, Stanford University, 443 Via Ortega, Stanford, CA 94305 USA; 3 School of Medicine, Stanford University, 291 Campus Drive, Stanford, CA 94305 USA; 4 Department of Engineering, Swarthmore College, 500 College Avenue, Swarthmore, PA 19081 USA

**Keywords:** Clinical immersion, Needs finding, Biodesign innovation process, Block-plan course, Curriculum development, Undergraduate engineering education, Experiential learning theory, Constructivist learning theory

## Abstract

Many undergraduate educational experiences in biomedical design lack clinical immersion-based needs finding training for students. Convinced of the merits of this type of training for undergraduates, but unable to offer a quarter-long course due to faculty and administrative constraints, we developed an accelerated block-plan course, during which students were dedicated solely to our class for 3 weeks. The course focused on the earliest stages of the health technology innovation process—conducting effective clinical observations and performing comprehensive need research and screening. We grounded the course in experiential learning theory (with hands-on, collaborative, and immersive experiences) and constructivist learning theory (where students integrated prior knowledge with new material on need-driven innovation). This paper describes the design of this intensive block-plan course and the teaching methods intended to support the achievement of five learning objectives. We used pre- and post-course surveys to gather self-reported data about the effect of the course on student learning. Despite the accelerated format, we saw statistically significant gains for all but one sub-measure across the learning objectives. Our experience supports key benefits of the block-plan model, and the results indicate that specific course design choices were effective in achieving positive learning outcomes. These design decisions include (1) opportunities for students to practice observations before entering the clinical setting; (2) a framework for the curriculum that reinforced important concepts iteratively throughout the program; (3) balanced coverage of preparation, clinical immersion, and need research; (4) extensive faculty and peer coaching; and (5) providing hands-on prototyping opportunities while staying focused on need characterization rather than solution development. Based on our experience, we expect that this model is replicable across institutions with limited bandwidth to support clinical immersion opportunities.

## Introduction

One compelling way to spark interest in biomedical engineering and other career paths at the intersection of technology and medicine is to provide students with immersive opportunities to observe the delivery of patient care and identify important unmet needs for themselves [1]. Clinical immersion programs also have been credited with better preparing students for positions in industry by giving them a real-world understanding of the user needs that new health technologies are intended to address [1]. Another significant benefit is that clinical immersion experiences create the chance to learn the importance of rigorous need research and screening/filtering, following shadowing, to translate raw observations into meaningful unmet needs that represent promising technology innovation opportunities [2].

For years, clinical immersion opportunities were generally reserved for graduate-level students and post-graduate learners due to the high administrative barriers associated with facilitating medical clearances and fulfilling requirements to protect patient privacy [3–5]. However, over the last decade, an increasing number of programs have begun offering undergraduates access to the clinical environment, in part, at the urging of students eager for clinical exposure. As Kotche et al. describe [1], these programs vary in duration from hour-long shadowing sessions to year-long experiences; however, those most prevalent in the literature are paid, summer internships or courses up to 10 weeks in duration, offering students as much as 20–30 hours in the clinical environment per week (> 150 total hours).

Our group was interested in offering a clinical immersion experience for undergraduate learners, but we were unable to offer a quarter-long (10-week) course due to faculty and administrative constraints, so we sought to design an accelerated experiential learning program to address this unmet need. Our primary goal was to teach the earliest stages of the need-driven innovation process, which include conducting effective clinical observations and performing comprehensive need research and screening. We also had an interest in exciting students about careers in healthcare and health technology innovation; however, we did not explicitly study this secondary objective for the purposes of this paper.

We became aware of the opportunity to offer a block-plan course [6], during which students dedicate themselves to a single class for a 3-week period, through our institution’s Sophomore College program. The Sophomore College is an immersive, fulltime residential program for rising sophomores during the 3-week intersession period between the summer and fall quarters. The courses offered as part of the program are intended to provide deep dives into student areas of interest and provide hands-on learning within and outside the classroom, while further enabling undergraduate learners to build confidence and gain experience forging relationships with faculty and peers. We hypothesized that an accelerated block offering would enable us to achieve our goals and committed to piloting the approach. The purpose of this paper is to describe the 3-week course, share results of the self-reported student data, and highlight the key course design decisions that we believe were most central to generating positive learning outcomes.

## Program Description

Like many block-plan programs, the Sophomore College model is intended to support reduced class sizes, eliminate competing priorities for students, and deepen learning experiences [7, 8]. In the year our class was offered, Sophomore College courses were permitted 13 days of instruction over 3 weeks. Students could enroll in only one course during the intersession period and were expected to devote 25–30 h per week to in-class activities and homework. They had no other commitments during this time except a few social/networking activities organized by the Sophomore College program. Class sizes were capped at 14 students. In terms of curriculum, we were instructed to emphasize experiential learning (more than didactic instruction) and to include activities that promote student interactions with faculty, as well as with one another.

Best practices in block-plan course development indicate that, to support effective learning, instructors simply cannot condense a traditional course curriculum into an accelerated timeframe. Intensive courses require a variety of teaching methods to maintain student motivation and interest [8, 9]. Moreover, faculty needs to be selective about what content is (and is not) included, with greater focus placed on the process of learning, time management, and critical thinking skills. Didactic instruction should be delivered in creative formats such as “short bursts” that are interspersed with individual exercises, group work, and hands-on activities [8].

We also grounded our course design decisions in experiential and constructivist learning theories [10]. Application of experiential learning theory provides opportunities for hands-on, collaborative, and immersive experiences that drive the generation of student questions and understanding (e.g., supporting small and large group work, placing an emphasis on coaching). The constructivist framework provides for unique learning experiences as students apply prior knowledge sets to the processing and integration of new material, such as lessons about the early-stage need-driven innovation process (e.g., performing and evaluating clinical observations).

With the structure of the Sophomore College, block-plan guidelines, and relevant learning theories in mind, we defined five core-learning goals for our course as shown in Table 1. Then, in support of these learning objectives, we divided the course roughly into thirds (Table 2).

**Table 1:** Course learning objectives (LOs)

After taking this course, students will be able to…
1. Understand and apply the fundamentals of the need-driven biodesign innovation process
2. Perform effective first-hand clinical observations
3. Draft meaningful need statements (e.g., that include a clear problem, population, and outcomes and show alignment among those three elements)
4. Conduct need research and use it to iteratively improve need statements
5. Use need research to competitively screen needs

**Table 2:** Highlights of course curriculum with linkages to learning objectives

Learning objectives	Week 1 course content	Week 2 course content	Week 3 course content
1. Understand and apply the fundamentals of the need-driven biodesign innovation process	Workshop: hands-on introduction to biodesign innovation process Panel of innovators: need-driven innovation in practice		
2. Perform effective first-hand clinical observations	Lecture: preparing for and performing observations Lecture: conduct in the clinical environment Assignment: perform background research for sim center exercise Exercise: practice clinical observations in sim center Assignment: perform background research for clinical observation shifts in week 2	Activity: perform clinical observations (three shifts per student) Discussions: small-group debriefs with faculty after each observation shift Assignment: record 10 compelling observations after each observation shift	
3. Draft meaningful need statements (e.g., that include a clear problem, population, and outcomes and show alignment among those three elements)	Workshop: translating observations from sim center into preliminary need statements	Assignment: write preliminary need statements for 3 compelling observations after each observation shift Assignment: choose 3 most compelling observations overall to work on in class	
4. Conduct need research and use it to iteratively improve need statements	Lecture: performing need research Case study with exercise: using the need research worksheet	Exercise: perform preliminary need research on top 3 needs (and refine need statements) Workshop: present top 3 need statements/research to a peer and discuss; perform additional research Workshop: need research “jigsaw” to reinforce key research concepts	Exercise: present updated need statement/research for top need to faculty; identify and address gaps Lecture: performing validation interviews Activity: conduct at least 2 validation interviews for top need Workshop: prototyping the need Final presentation: present project to faculty with go/no-go recommendation on taking need forward based on research completed to date
5. Use need research to competitively screen needs	Lecture:/case study: need filtering	Exercise: present top 3 need statements/research to faculty and filter to a top need	

### Week One: Preparing for Clinical Observations

The first week (4 days) was spent preparing students for clinical immersion. This started by exposing them to the benefits of need-driven innovation and the steps in the biodesign innovation process through a hands-on, team-based workshop. We also convened a panel of innovators to share stories of how starting with the need aided them in their entrepreneurial efforts. Another area of focus was on how to perform observations and conduct oneself in the clinical environment. This involved didactic instruction, including the introduction of multiple frameworks (e.g., what “clues” might signal unmet needs when shadowing), tips on notetaking, and guidelines on behavior (note that the students completed HIPAA training prior to beginning the course). In addition, we partnered with one of our institution’s clinical education/simulation centers to provide the students with observation “practice.” For this workshop, clinicians and volunteers play-acted two scenarios: (1) a situation where post-operative complications result in the need for emergency corrective surgery, and (2) the procedure of placing a central line. All students observed the scenarios together, taking notes on potential unmet needs. Then, after each one, we debriefed on the experience and used examples from their notes to demonstrate how to translate an observation into the first draft of a need statement. The remainder of the week was spent learning about need research and need filtering through a combination of lectures, case examples, and hands-on activities.

### Week Two: Performing Clinical Observations, and Research and Screening Needs

The top priority in week two (5 days) was on performing observations. Students observed in pairs, and each duo was given the opportunity to spend approximately four hours in three different clinical environments across 3 days: (1) the emergency department, (2) an outpatient clinic, and (3) an operating room. Given the limited time available for shadowing, our intent was to expose them to the widest range of clinical encounters possible. After each day’s observation experience, the students debriefed with a member of the teaching team to allow them to reflect on what they had seen. As homework, they individually went through their notes to identify their 10 most compelling observations and draft need statements for their top three. Compelling observations, we explained to the students, reflect scenarios that seem to have an objectively detrimental impact to patients and/or providers and the current solutions, if any exist, have significant shortcomings. Over time, the students would confirm whether or not observations that seemed compelling at first represent meaningful needs worth pursuing by doing deeper need research (i.e., is it a generalizable issue across a range of geographies and healthcare settings, are there stakeholder issues that may make it extremely challenging to effectively implement a solution, etc.).

Students spent the rest of the week performing this research and screening down to a lead project. This was done with the help of worksheets to aid the student in focusing their research. This guided approach to need research was intended to expose students to the importance of defending and validating a clinical need with real evidence. We focused their efforts around the “four pillars” of need research per the biodesign innovation process, which are (1) understanding the problem or fundamentals of the disease state/health condition; (2) assessing the impact of the problem in terms of the size of the affected population and related economic cost; (3) identifying what existing solutions already exist and their relative strength and weaknesses; and (4) defining which stakeholders are most impacted by the need and how they may determine or influence the adoption of a new solution**.** [See https://biodesignguide.stanford.edu/toolkit/becoming-an-expert-in-your-need-through-research/ for an example need research worksheet.] Faculty met with students one-on-one or in pairs to qualitatively assess their research findings and provide feedback on gaps or questions/topics they should pursue further in validating their top need.

### Week Three: Researching, Validating, and Prototyping Top Needs, and Final Presentations

Week three (4 days) was spent more deeply understanding each student’s lead project. A highlight was an activity focused on prototyping the need. Students were prompted to ask questions about their need area, and then design and build models and tests to address those questions. For example, one student who was working on an unmet need related to chronic wound healing had these questions: (1) what does the structure of different stages of wounds look like in 3-dimensional space? and (2) how does it feel for patients to care for these wounds? She built an anatomical model of a wound with different materials representing different layers of the ulceration. Through this exercise, she gleaned insights around wound architecture and shape, differences in the physical properties of the tissue layers of the wound, and greater empathy for the patient. [Learn more about prototyping the need at https://biodesignguide.stanford.edu/wp-content/uploads/2022/07/Examples-of-Prototyping-the-Need.pdf.] Other experiential activities included validation interviews with multiple care providers and peer-review practice for final presentations. The validation interviews, as a form of primary research, were particularly important in terms of teaching students to use first-hand data collection techniques to address gaps in information available through the literature and/or to test critical assumptions made based on their secondary research. On the last day of class, students presented their lead projects to one another and the teaching team.

The course consisted of other elements intended to further develop student skills and to facilitate increased engagement, confidence, and enthusiasm for health technology innovation. Specifically, in parallel with the core curriculum, we hosted a career discussion and orchestrated networking opportunities with alumni of our training programs to help students consider different pathways at the intersection of medicine and engineering. Instructors also participated in extracurricular activities to get to know students personally and help them overcome perceived barriers between faculty and students.

## Methods

To assess the effect of the course on student learning, we developed a survey to administer before and after the course. The purpose of the survey was to collect self-reported data on student demographics (pre) and student perceived proficiency (pre and post) related to our five learning objectives. All questions were mandatory for students to complete. The demographic data were collected for grant reporting purposes but were not included in the analysis for this paper.

For each learning objective, we defined from one to four sub-measures that would enable us to gauge student proficiency. We then translated those sub-measures into 13 quantitative questions with response options that corresponded to 1–5 values on a Likert scale, with 1 being low and 5 being high. In addition, we asked one free text response question. See Table 3 for a summary of the sub-measures, questions, and response options by learning objective.

**Table 3:** Sub-measure, questions, and response options for each course learning objective

Sub-measures	Survey question	Response options
LO1—understand the fundamentals of the need-driven biodesign innovation process		
Know the difference between tech-push and need-pull innovation	1. How well do you understand the difference between tech-push and need-pull innovation?	1. I have never heard of either 2. I have heard of them but don’t really understand the difference 3. I understand the basic difference between them 4. I can explain the difference between them 5. I can explain the difference between them and give real-world examples
Articulate the three phases of the biodesign innovation process	2. How familiar are you with the biodesign innovation process?	1. I have never heard of it 2. I have heard of it, but don’t really understand what it is 3. I understand the basic approach 4. I know the biodesign innovation process and can explain the three phases 5. I have enough understanding/experience to practice the biodesign innovation process on my projects
Articulate the three components of the need statement (problem, population, and outcome)	3. Do you know what a need statement is?	1. I have never heard of it 2. I have heard of it, but don’t really understand what it is 3. I understand the basics of the need statement 4. I know what it is and can explain the three components of the need statement 5. I understand how to develop and refine a need statement by experimenting with different variations
Explain what need criteria are and why they’re important	4. Do you know what need criteria are?	1. I have never heard of them 2. I have heard of them, but don’t really understand what they are 3. I understand the basics of what need criteria are 4. I know what they are and can explain how and why they’re used 5. I understand how to develop objective and measurable need criteria
LO2—Perform effective first-hand clinical observations		
Comfortable talking to physicians and other care providers	5. How comfortable do you feel talking with physicians and other care providers?	1. Uncomfortable 2. Slightly uncomfortable 3. Neutral 4. Comfortable 5. Very comfortable
Understands responsible conduct for an observer in a clinical environment	6. How well do you understand how to behave responsibly when conducting observations in a clinical environment?	1. I have no idea how to behave responsibly as an observer 2. I can guess how to behave responsibly as an observer 3. I understand the basics of how to behave as an observer 4. I understand how to behave responsibly as an observer and can explain this to others 5. I understand how to behave responsibly as an observer and feel comfortable observing in any clinical environment
Able to perform effective first-hand clinical observations	Please list 5 one-word “clues” you would watch for when performing observations in an effort to identify compelling unmet health-related needs	[Unstructured text entry] [Note: This question is not numbered because it is not included with the quantitative data summarized in Figure 1]
Confident in ability to identify interesting innovation opportunities through observations	7. How confident are you in your ability to identify interesting innovation opportunities through observations?	1. Unconfident 2. Somewhat unconfident 3. Neutral 4. Confident 5. Very confident
Recognize that every observation may not represent a compelling unmet need	8. To what extent do you agree with the statement “every observation represents a compelling unmet need”?	1. Strongly disagree 2. Disagree 3. Neutral 4. Agree 5. Strongly agree
LO3—Draft meaningful need statements (e.g., that include a clear problem, population, and outcomes and show alignment among those three elements)		
Construct need statements with the requisite parts (PPO)	9. How comfortable do you feel constructing a need statement (with all of its requisite parts) based on a clinical observation?	1. Uncomfortable 2. Slightly uncomfortable 3. Neutral 4. Comfortable 5. Very comfortable
LO4—Conduct need research and use it to iteratively improve need statements		
Articulate what the four pillars are and where to find information on them	10. Do you know what the four pillars of need research are?	1. I have never heard of them 2. I have heard of them, but don’t really understand what they are 3. I understand the basics of the four pillars of need research 4. I understand the four pillars and know what to look for when conducting need research in each area 5. I understand the four pillars, know what to look for when conducting need research, and can explain where/how to find information on each pillar
Create questions and lead validation interviews	11. How comfortable are you preparing for and conducting validation interviews?	1. Uncomfortable 2. Slightly uncomfortable 3. Neutral 4. Comfortable 5. Very comfortable
Able to advance understanding of a need through prototyping	12. To what extent do you understand how to use prototyping to advance your understanding of an unmet health-related need?	1. I have never heard of this 2. I have prototyping experience, but don’t know how to use it to advance my understanding of an unmet health-related need 3. I understand the basics of using prototyping to advance my understanding of an unmet health-related need 4. I know how to do this and can explain it to others 5. I know how to do this and have used it on a project to advance my understanding
LO5—Use need research to competitively screen needs		
Able to describe how filtering works when dealing with many needs	13. To what extent do you understand an objective approach for screening and prioritizing unmet health-related needs to decide on a top project?	1. I have never heard of this 2. I have heard of this, but don’t really understand what it is 3. I understand the basics of using an objective approach for screening and prioritizing unmet health-related needs 4. I understand how to do this and can explain it to others 5. I understand how to do this and have real-world experience using an objective approach for screening and prioritizing unmet health-related needs

Students completed the survey in Qualtrics (Provo, UT) under protocol 56713, approved by our university’s Institutional Review Board. We assigned the pre-course survey as homework before the first day of class. Students then completed the same survey on the last day of class, after making their final presentations.

Survey results were compiled and anonymized by a staff member who was not a member of the course teaching team.

## Statistical Methods

We analyzed the survey data using a two-tailed, paired Wilcoxon Signed-Ranked Test ($\alpha \le 0.05$) to determine statistical significance for all Likert-scale questions. For the free text question, we listed all of the responses, tallied the frequency of specific responses, and then compared the pre-course and post-course survey data.

## Results

All 12 students completed the pre- and post-course surveys and consented to be part of the study. We found statistically significant improvements in 12 of 13 quantitative questions.

There was a 100% or greater change between the pre-course and post-course survey median values for seven of 13 questions as shown in Table 4.

**Table 4:** Survey questions with 100% change or greater percent change between pre-course and post-course median score

Learning objective	Question	% Change median (interquartile range)
LO1	Q1—How well do you understand the difference between tech-push and need-pull innovation?	300% (56%, 400%)****
LO1	Q2—How familiar are you with the biodesign innovation process?	125% (67%, 150%)****
LO1	Q3—Do you know what a need statement is?	150% (67%, 150%)****
LO1	Q4—Do you know what need criteria are?	125% (92%, 212%)***
LO4	Q10—Do you know what the four pillars of need research are?	400% (262%, 400%)******
LO4	Q12—To what extent do you understand how to use prototyping to advance your understanding of an unmet health-related need?	150% (67%, 250%)***
LO5	Q13—To what extent do you understand an objective approach for screening and prioritizing unmet health-related needs?	125% (67%, 400%)***

Statistical significance determined using Wilcoxon signed-ranked test, *< 0.05, **< 0.005, ***< 0.0005, ****< 0.00005, *****< 0.000005, ******< 0.0000005

While still statistically significant, the data for five questions had a change of less than 100% between the pre-course and post-course medians. These included, “How comfortable do you feel talking with physicians and other care providers?” (median: 25%; interquartile range: 0% to 27%); “How well do you understand how to behave responsibly when conducting observations in a clinical environment?” (46%; 19% to 75%); “How confident are you in your ability to identify interesting innovation opportunities through observations?” (58%; 25% to 75%); “How comfortable do you feel constructing a need statement (with all of its requisite parts) based on a clinical observation?” (83%; 33% to 188%); and “How comfortable are you preparing for and conducting validation interviews?” (58%; 25% to 125%).

The one question that was not statistically significant was, “To what extent do you agree with the statement ‘every observation represents a compelling unmet need’?” (12%; 0% to 27%).

**Fig. 1:**
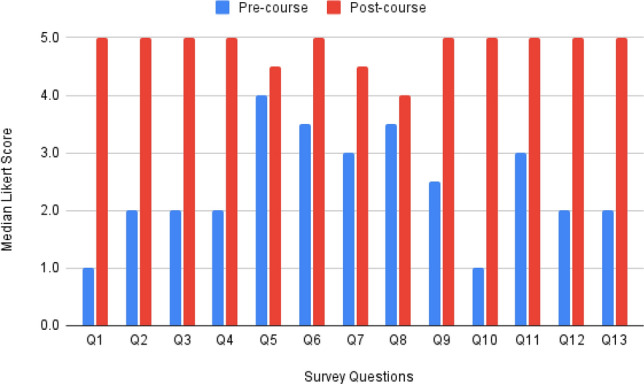
The figure depicts changes in median scores across the 13 quantitative questions in the pre- and post-course surveys. Student scores were higher at statistically significant levels for all but one question (#8) on the post-course survey (red bars) compared to the pre-course survey (blue bars). The question numbers correspond to the survey questions listed in Table 3

The changes in pre-course and post-course median scores are summarized in Fig. 1.

## Discussion

Overall, we found that the block-plan model was an effective approach, enabling students to achieve the course learning objectives with statistically significant gains for most sub-measures across all learning objectives.

Interestingly, while students considered the clinical observations to be a highlight of the course, the greatest perceived learning improvements were linked to students’ understanding of the innovation process (e.g., understanding the difference between tech-push and need-pull innovation, need statements, the four pillars of need research, need criteria, and need filtering). This could be because the course was offered to rising sophomores who had not yet been exposed to these concepts through other courses, so the difference in their understanding pre- and post-course was substantial. The gains were comparatively modest on student comfort levels interacting with physicians and other care providers, understanding conduct in the clinical environment, identifying interesting innovation opportunities through observations, and preparing for and conducting validation interviews. Because the pre-course ratings for these questions were higher than most other questions, we hypothesize that our students generally have relatively high comfort/confidence levels in new situations, particularly in healthcare as many of the students tend to be interested in pursuing careers in medicine. In addition, we informally learned that some had previously done shadowing, and a couple had substantial experience interacting with the healthcare system as patients or family members of patients.

For the Likert-scale question that did not have statistically significant results (“To what extent do you agree with the statement ‘every observation represents a compelling unmet need’?”), we realized during data analysis that we unintentionally inverted the rating scale, with the most positive response (“strongly disagree”) assigned a 1 and the least positive response (“strongly agree) assigned a 5. As a result of this error, we considered the data for this question to be inconclusive and will address this in future surveys.

For the fill-in-the-blank question (“Please list 5 one-word ‘clues’ you would watch for when performing observations in an effort to identify compelling unmet health-related needs”), the variability of the word choice was high, and we similarly were not able to draw any clear conclusions. However, we noted that, in the post-course responses, students were more likely to list words that were part of the observation frameworks taught in class and there was more frequent overlap in the words the students chose.

Self-reported data have inherent limitations. In future studies, we aim to more objectively assess student knowledge levels before and after the class by including instructor assessments of student performance according to a rubric that is under development. To the best of our knowledge, evaluation tools for assessing the performance of novice students aligned with the learning objectives of this intensive curriculum do not exist. Wieman et al. recommend first observing ‘experts’ performing a skill, and then extracting features of their skillset as the basis of a rubric [11, 12]. In the absence of student experts at this introductory level, we are pursuing a modified Wieman approach. Specifically, we have observed/documented what students do under the guidance of supportive instruction and are now working to identify the features that will serve as the basis of a trial rubric that we will pilot during the next offering of the course.

Another limitation is that we are unable to say how much more students would gain through a longer program with more hours of clinical shadowing and additional time for need research and screening. In the absence of a longer clinical immersion program for undergraduate students at our institution, we do not have a readily available way to make a direct comparison. However, this is an issue that we will consider for further study, perhaps in collaboration with another university.

Beyond the results of the pre- and post-course surveys, we observed other benefits linked directly to the block-plan model. First, scheduling shadowing opportunities was simplified since we had open access to student’s time and did not have to work around conflicts created by other classes. Second, because we could devote 25–30 h per week to the curriculum, we had the opportunity to build substantial blocks of work time into the course design within and outside of class to enable students to go deeper into the materials than they might when managing multiple courses. Third, the small class size enabled us to get to know students individually so that we could explicitly explore each one’s career interests, answer questions about next steps on their path (e.g., decisions about what to major in, ways to gain relevant experience), and introduce them to others who had pursued similar career choices. As a result, students expressed great excitement about careers at the intersection of medicine and engineering. In the written course evaluations conducted by the university, students shared comments such as, “The most important thing I am taking away from my experience are new insights towards my career;” “This [course] has greatly impacted how I imagine my future;” and “The needs finding process was an amazing perspective shift for me, one that I know I'll be keeping in mind for the rest of my career.”

There were five other course design choices that we believe were central to supporting the positive learning outcomes realized through the course. While these design choices were well suited to the block-plan format, and many are supported by experiential and constructivist learning theory, they are equally relevant when planning a traditional quarter- or semester-long course and can be adapted accordingly.

1. *Practicing in controlled environment before doing observations in clinical setting* When teaching preparation for observations, the exercise in the simulation center was a highlight for students because it shifted their learning from theoretical to applied. They benefitted from the opportunity to practice looking for “clues” that might signal unmet needs, using different notetaking techniques, and staying focused during fast-paced and slow-moving clinical encounters (the post-operative and central line simulations, respectively). The exercise further provided faculty with a rare opportunity to see the same things, at the same time as the students observed them, making our debrief discussions more concrete and actionable. Students also told us that the ability to translate the shared observations into preliminary need statements as a group was a useful part of the activity. So, while the simulation center workshop required substantial time and effort to orchestrate (as well as a modest fee, paid to the simulation center), it proved to be invaluable.
2. *Balanced coverage of preparation, clinical immersion, and need research/screening* As noted, the course included just 3 days of clinical immersion (out of a total of 13 instructional days) to allow significant time for preparation and practice, as well as downstream need research, screening, and validation. While these surrounding activities are not as exciting to students, they are critically important to identifying a worthwhile focus for an innovation project. Through the balanced structure of the curriculum, as well as a number of specific lectures and activities focused on need research and validation, we emphasized this to students. As a result, they experienced how rigorous preparation and disciplined follow-through surrounding observations greatly improve one’s chances of deciding on a promising project area. As one student described in their course evaluation, “The value of heavy needs research before solution brainstorming was [emphasized] because, in healthcare, making a solution that doesn't meet the correct need costs years and huge amounts of money.” And because the students observed in three different environments—the emergency department, an outpatient clinic, and an operating room—they gained diverse clinical exposure despite the short duration.
3. *Iterative work at progressively deeper depth* Throughout the course, we introduced core concepts and then gave students multiple opportunities to apply them at an increasing level of detail. For example, on the first day of class, we took the students through a three-hour workshop on need finding and need screening. Working in teams, they rapidly learned and practiced the key steps in those stages of the process on a simulated innovation project before we asked them to apply that knowledge to their own work. Similarly, with need research—one key element of need screening—we took the students through multiple rounds of learning and application. In week one, they had a didactic overview of need research and practiced it through a brief simulated exercise. In week two, they performed high-level need research across multiple needs as a mechanism for deciding on a top project. Then, in week three, they went even deeper to characterize their top need for their final presentation. Through this cycle, the students gained progressively greater mastery of need research concepts.
4. *Stopping short of solution generation, but finding other ways to incorporate prototyping* Some clinical immersion programs described in the literature incorporate solution generation as a follow-on to observations and need statement development [13]. This is understandable since engineering students and other aspiring health technology innovators generally are eager to problem solve and do hands-on building. However, jumping ahead to these activities before students have done rigorous need research and screening is a shortcut in a disciplined innovation process and contradictory to our learning objectives. By developing the aforementioned workshop on prototyping the need, the students were able to learn about the identified need through a hands-on building opportunity. This added excitement to the student’s need research and satisfied their desire to be creative and build prototypes without moving prematurely into a solution mindset. As the student who developed the anatomical model of a wound to advance her thinking around unmet needs related to chronic wounds described, “I was initially really focused on thinking ahead to how I would come up with a novel innovation in the area of chronic wounds. But I realized that prototyping aspects of the need can be just as impactful because it gives you a new perspective on the problem you’re trying to solve.”
5. *Extensive faculty and peer coaching* One additional design decision to highlight is the emphasis we placed on personalized coaching. The combination of the small class size and number of available instructional hours enabled us to build-in coaching activities at each step of the innovation process, and in support of each learning objective. These sessions varied in nature but included 1:1 and small-group meetings with faculty members, as well as peer-review exercises. For example, after each day of clinical immersion, students spent 20–30 min debriefing with a faculty member, reflecting on what they observed, and asking questions. The meetings helped the students make sense of their clinical experience while allowing faculty to track their progress, reinforce key learnings, and build a personal connection with each student. Similar sessions were held at multiple points during need research and screening, enabling us to help students overcome research challenges and build confidence in their decisions as they moved from multiple needs to a top project. Peer reviews were particularly useful in helping students prepare for and practice their final presentations.

## Conclusion

Clinical immersion programs that span an academic quarter or semester will continue to play an important role in biomedical engineering education, yet they are resource intensive and inaccessible in many contexts. The block-plan intersession approach presented here provides an alternate model that achieves significant learning outcomes in an accelerated timeframe. This model also has the potential to reach more students, especially at those institutions unable to offer more extensive shadowing opportunities.

## Data Availability

Not applicable.
